# Immunization‐related complex regional pain syndrome: A systematic review of case reports

**DOI:** 10.1002/pcn5.70041

**Published:** 2024-12-10

**Authors:** William K. Copenhaver, Brandon J. Goodwin, Alexa Simonetti, Kunal P. Shah, Nicholas J. Averell, David F. Lo, Richard T. Jermyn

**Affiliations:** ^1^ Futures Forward Research Institute Toms River New Jersey USA; ^2^ Department of Biology Rutgers, The State University of New Jersey New Brunswick New Jersey USA; ^3^ Department of Medicine Rowan University School of Osteopathic Medicine Stratford New Jersey USA

**Keywords:** complex regional pain syndrome (CRPS), immunization, pain

## Abstract

**Aim:**

Vaccines have been shown to have the highest efficacy in preventing infectious diseases through their ability to induce immunological memory against pathogens. An adverse reaction to a vaccine is an unexpected medical occurrence following immunization. Complex regional pain syndrome (CRPS) is a disease that has undergone much controversy regarding its onset post‐vaccination. This systematic review aims to evaluate cases of CRPS post‐vaccination to better understand the manifestation of the disease and its potential association with vaccines.

**Methods:**

A systematic review of case reports was conducted employing the PRISMA 2020 guidelines. Outcomes of interest include type of vaccination, patient age, patient sex, time to symptom onset, and medical history including but not limited to previous autoimmune diseases, psychological illness, physical tissue trauma, and neurological disease.

**Results:**

Initial querying of the five databases yielded 404 articles. Following a thorough review of articles, only 14 remained, comprising 18 cases. Studies included cases of CRPS development following tetanus, hepatitis B, hepatitis A, rubella, influenza, tetanus–diphtheria, human papillomavirus, and COVID‐19 vaccine administration.

**Conclusion:**

The limitations of evidence used in this study highlight the need for a greater output of higher‐level evidence in the form of controlled trials and retrospective studies to help further elucidate the connection between vaccine use and the development of CRPS in patients. Currently, vaccines continue to be safe for global public use.

## INTRODUCTION

Vaccines have been shown to have the highest efficacy in preventing infectious diseases through their ability to induce immunological memory against pathogens.^1^ There are four types of vaccines: live attenuated, inactivated, subunit, and toxoid.^2^, ^3^, ^4^ All vaccine types serve the same purpose of initiating an immune response to produce antibodies and provide long‐term immunity and defense against a specific pathogen. An adverse reaction to a vaccine is an unexpected medical occurrence following immunization and may occur as the direct result of immunization or an indirect result not associated with the immunization process. Adverse reactions can manifest as immune‐ or non‐immune‐mediated reactions, have an immediate or delayed presentation, and can affect the local area or spread systemically.^5^, ^6^

Complex regional pain syndrome (CRPS) is a disease that has undergone much controversy regarding its onset post‐vaccination. CRPS is a rare inflammatory and neuropathic condition that develops after sustaining an injury or undergoing surgery to a limb, most commonly involving the distal radius.^7^ The incidence of CRPS is estimated to be 26.2 per 100,000 person‐years, displaying a higher prevalence in females at a ratio of 4:1 when compared to males.^8^, ^9^, ^10^ Patients with CRPS present with debilitating sensations along with motor, sensory, and autonomic defects in the distal part of the affected limb.^11^ Symptoms include painful sensations in the forms of allodynia or hyperalgesia. Changes to the skin of the affected area result in edema, abnormal blood flow, and asymmetric sweating, as well as dystonia, and neuropsychological changes, such as distorted body representation and lateralized spatial cognition deficits.^12^ There are two types of CRPS: Type 1 is more common and lacks an identifiable nerve lesion, while Type 2 is defined by the presence of a nerve lesion or injury.^13^ Clinical diagnosis is made using the established guidelines for diagnosing CRPS known as the Budapest Criteria (Table 1).^14^ The Budapest Criteria are generally preferred over the original International Association for the Study of Pain (IASP)^15^ criteria and are accepted by the IASP due to greater validity and specificity.^14^, ^16^

**Table 1:** Budapest diagnostic criteria.

**All of the following statements must be met:**		
1. The patient has continuous pain that is disproportionate to the inciting event		
2. The patient has at least one sign in two or more categories		
3. The patient reports at least one symptom in three or more categories		
4. No other diagnosis can better explain the signs and symptoms		

**Category:**	**Sign: recognized by clinician**	**Symptom: Reported by patient**
1. Sensory	Allodynia and/or hyperalgesia	
2. Vasomotor	Temperature asymmetry and/or skin color changes/asymmetry	
3. Sudomotor/Edema	Edema and/or sweating changes/asymmetry	
4. Motor/Trophic	Decreased range of motion and/or motor dysfunction and/or trophic changes (hair/skin/nails)	

In March 2013, the development of CRPS became a significant point of controversy following human papillomavirus (HPV) vaccination in Japan. Subsequently, numerous other cases came to light, totaling 17 reported cases (10 from Japan and five from the United Kingdom). In response, the Japanese ministry announced a temporary suspension of HPV vaccination pending further re‐evaluation by GlaxoSmithKline (GSK) and Merck, the sole manufacturers of the HPV vaccines. The corresponding reporting rates were 0.08 per 100,000 doses administered in Japan. A meticulous safety analysis conducted by GSK revealed that out of the 17 cases, only four met the diagnostic criteria for CRPS. Despite these findings, a careful analysis was performed, ultimately leading to the conclusion of a non‐significant relationship between CRPS and HPV vaccination.^17^

However, this suspension had significant consequences. The halt in HPV vaccination resulted in a drastic decline in the HPV vaccination rate in Japan, plummeting from 70% to 1%, marking one of the most dramatic falls ever recorded. This 7‐year suspension made cervical cancer one of the leading causes of mortality and a highly prevalent cause of cancer among women aged 15–39 years.^18^ In the USA, a reporting analysis was conducted in 2015 by searching MedDRA terms in the Vaccine Adverse Event Reporting System (VAERS), where 0.09% of CRPS cases were identified. It is important to note that using such databases can significantly impact drawing conclusions due to the potential for biases, such as over‐reporting, under‐reporting, biased reporting, or inconsistent reporting, all of which can lead to flawed findings. Suggestions have been made that post‐vaccination CRPS could be attributed to minor injection trauma during vaccination. However, both the comprehensive analysis by Huygen et al. and the VAERS database indicate that such adverse effects are rare.^19^

Somatic symptom disorder is a stigmatized condition that revolves around preoccupation with a symptom (or symptoms) that has no medical explanation. The somatic complaint must cause significant distress and can disrupt functioning. Pain can be the predominate symptom reported.^20^ As Type 1 CRPS is idiopathic, it can be misdiagnosed as a somatoform disorder and vice versa,^21^ though this has not been systematically studied. Psychiatrists, neurologists, and pain physicians alike should consider somatoform disorders and CRPS in appropriate clinical scenarios. After a seemingly innocuous stimuli, like an intramuscular injection, development of chronic pain could be considered as a somatoform disorder, unless specific criteria for CRPS are met.^14^

Given the ambiguity surrounding the onset and symptoms of CRPS post‐vaccination, the possibility that CRPS can manifest as an adverse reaction to immunization is possible but not well understood. The understanding that adverse reactions can be a direct or indirect result of immunization may shed light on immune‐mediated reactions, which can present variably, either locally or systemically.^5^ This systematic review aims to evaluate case reports and case series of CRPS post‐vaccination in an attempt to better understand the manifestation of the disease and its potential association with vaccines.

## METHODS

A systematic review of case reports was conducted employing the PRISMA 2020 guidelines.^22^

### Inclusion criteria

To be included in the analysis, articles must contain primary patient data on CRPS following a vaccination. Types of articles included in the analysis are case reports and case series. Outcomes of interest include type of vaccination, patient age, patient sex, time to symptom onset, and medical history including but not limited to previous autoimmune diseases, psychological illness, physical tissue trauma, and neurological disease. Full‐text must be available either via inter‐library loan, journal, or publishing house.

### Exclusion criteria

To be excluded from analysis, articles must not contain information on CRPS following immunizations. Publications that were not case reports or series were excluded. Articles were not excluded based on their publication language, country of origin, or date. Gray literature was excluded from the analysis.

### Information sources and search strategy

Five databases were queried to investigate the occurrence of vaccinations/immunizations and the subsequent development of CRPS. PubMed, Web of Science, Cochrane, Scopus, and Embase were employed as the databases for this systematic review. The Boolean string employed in this analysis was the following: (‘complex regional pain syndrome’ OR ‘causalgia’ OR ‘reflex sympathetic dystrophy’ OR ‘complex regional pain syndromes’ OR ‘CRPS’) AND (‘vaccine’ OR ‘vaccination’ OR ‘immunization’). The initial database query was conducted in September 2023. Resulting articles were then uploaded to Rayyan.ai for duplicate detection and removal. Following removal, articles were then screened a second time manually to ensure accuracy.

### Study selection

**Figure 1:**
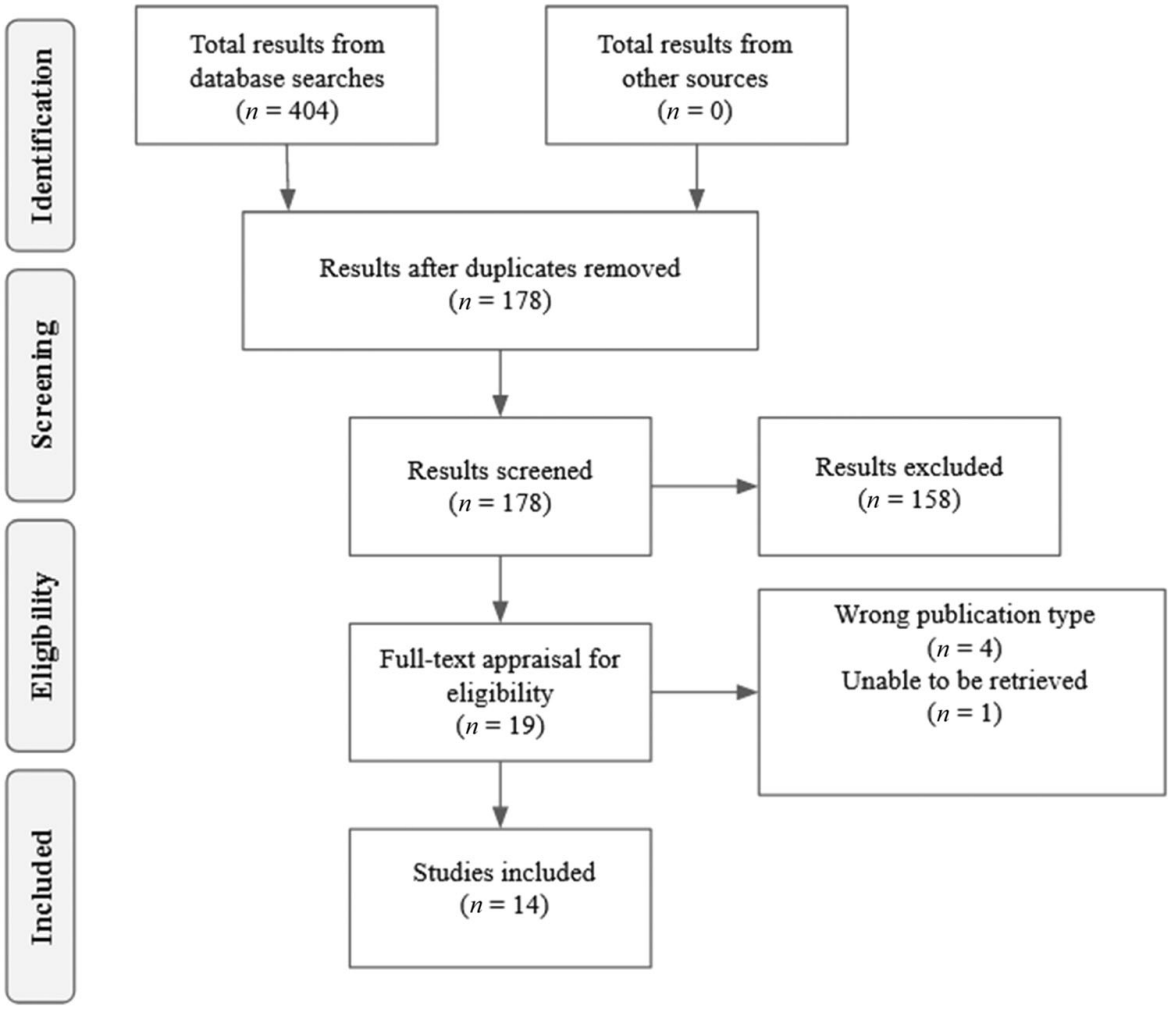
PRISMA flow diagram.

After duplicate detection and removal, articles were then appraised for title and abstract relevance and content. Articles were kept based on adherence to inclusion criteria, or failure to meet exclusion criteria. Accordingly, the remaining articles were then appraised for full text by two trained reviewers to ensure accuracy. If the two reviewers were split on an article, a third trained reviewer would read and judge the article to break the stalemate. The remaining articles then had their data extracted into a spreadsheet to be prepared for analysis. Studies not in English were translated via Google Translate for analysis, so long as information could still be extracted. The detailed PRISMA flow sheet is depicted in Figure 1.

### Data collection

The included articles subsequently had their data extracted. Data was extracted by two authors (Brandon J. Goodwin and William K. Copenhaver). Brandon J. Goodwin performed the first extraction and William K. Copenhaver checked for accuracy. Primary outcomes of interest included: type of vaccination, patient sex and age, and pertinent medical history. Articles were then sorted by type of vaccination and ordered by date. Data were prepared for qualitative analysis in narrative and table form.

### Article grading and methodological quality

Once an article has been deemed suitable for inclusion in the analysis, two trained reviewers examined them for quality and strength of evidence utilizing the Grading of Recommendations Assessment, Development, and Evaluation (GRADE) criteria.^23^ Each article was reviewed independently by authors William K. Copenhaver and Nicholas J. Averell. Results were then compared for independent congruity, differences were discussed, and a final appraisal of the strength of evidence was reached. Results are summarized in Table 2.1 and 2.2.

**Table 2.1:** GRADE summary. Pt 1.

Reference	Imprecision	Publication bias	Other	Overall GRADE
Bensasson^28^	No estimate of effect or intervention outcomes reported	Undetected	Not applicable due to nature of studies	Very low
Al‐Nesf et al.^29^	No estimate of effect or intervention outcomes reported	Undetected	Not applicable due to nature of studies	Very low
Pirrung^30^	No estimate of effect or intervention outcomes reported	Undetected	Not applicable due to nature of studies	Very low
Jastaniah et al.^26^	No estimate of effect or intervention outcomes reported	Undetected	Not applicable due to nature of studies	Very low
Genc et al.^31^	No estimate of effect or intervention outcomes reported	Undetected	Not applicable due to nature of studies	Very low
Kwun et al.^32^	No estimate of effect or intervention outcomes reported	Undetected	Not applicable due to nature of studies	Very low
Martínez‐Lavín^27^	No estimate of effect or intervention outcomes reported	Undetected	Not applicable due to nature of studies	Very low
Bilić et al.^33^	No estimate of effect or intervention outcomes reported	Undetected	Not applicable due to nature of studies	Very low
Seyrek et al.^34^	No estimate of effect or intervention outcomes reported	Undetected	Not applicable due to nature of studies	Very low
Koushik^35^	No estimate of effect or intervention outcomes reported	Undetected	Not applicable due to nature of studies	Very low
Raman^25^	No estimate of effect or intervention outcomes reported	High suspicion^a^	Not applicable due to nature of studies	Very low
Cho et al.^38^	No estimate of effect or intervention outcomes reported	Undetected	Not applicable due to nature of studies	Very low
Horisawa et al.^36^	No estimate of effect or intervention outcomes reported	Undetected	Not applicable due to nature of studies	Very low
Nogueira Pinto et al.^37^	No estimate of effect or intervention outcomes reported	Undetected	Not applicable due to nature of studies	Very low

Abbreviation: GRADE, Grading of Recommendations Assessment, Development, and Evaluation.

^a^ Raman^25^ reported an autobiographical case report in an open access journal.

**Table 2.2:** Quality of evidence rating for included studies, Pt. 2.

Reference	Study design	Risk of bias	Inconsistency	Indirectness
Bensasson^28^	Case report	Low	No – this was a single case	No – single case with no interventional analysis
Al‐Nesf et al.^29^	Case report	Low	No – this was a single case	No – single case with no interventional analysis
Pirrung^30^	Case report	Low	No – this was a single case	No – single case with no interventional analysis
Jastaniah et al.^26^	Case series	Low	No – this was a report of four cases with no statistical analysis	No – four cases with no interventional analysis
Genc et al.^31^	Case report	Low	No – this was a single case	No – single case with no interventional analysis
Kwun et al.^32^	Case report	Low	No – this was a single case	No – single case with no interventional analysis
Martínez‐Lavín^27^	Case series	Low	No – this was a report of two cases with no statistical analysis	No – two cases with no interventional analysis
Bilić et al.^33^	Case report	Low	No – this was a single case	No – single case with no interventional analysis
Seyrek et al.^34^	Case report	Low	No – this was a single case	No – single case with no interventional analysis
Koushik^35^	Case report	Low	No – this was a single case	No – single case with no interventional analysis
Raman25, a	Case report	High^a^	No – this was a single case	No – single case with no interventional analysis
Cho et al.^38^	Case report	Low	No – this was a single case	No – single case with no interventional analysis
Horisawa et al.^36^	Case report	Low	No – this was a single case	No – single case with no interventional analysis
Nogueira Pinto et al.^37^	Case report	Low	No – this was a single case	No – single case with no interventional analysis

^a^ Raman^25^ was downgraded two levels due to the autobiographical nature of the publication as there is risk of underreporting or overreporting of symptoms.

Methodological quality was assessed using a version of the Newcastle–Ottawa Scale devised by Murad et al., adapted for case series and case reports.^24^ The same trained reviewers followed the same procedure to reach an agreement on the methodological quality (also known as risk of bias) of the studies as was performed for the GRADE quality of evidence procedure. Results are summarized in Table 3.

**Table 3:** Risk of Bias Summary.

	Bensasson^28^	Al‐Nesf et al.^29^	Pirrung^30^	Jastaniah et al. ^26^	Genc et al. ^31^	Kwun et al.^32^	Martínez‐Lavín^27^	Bilić, et al. ^33^	Seyrek, et al^34^	Koushik^35^	Raman,^25^	Cho et al. ^38^	Horisawa et al.^36^	Nogueira Pinto et al.^37^
**Selection**														
1. Does the patient represent the center's overall experience?	Yes	Yes	Yes	Yes	Yes	Yes	Yes	Yes	Yes	Yes	Yes	Yes	Yes	Yes
**Ascertainment**														
2. Was the exposure adequately ascertained?	Yes	Yes	Yes	Yes	Yes	Yes	Yes	Yes	Yes	Yes	Yes	Yes	Yes	Yes
3. Was the outcome adequately ascertained?	Yes	Yes	Yes	Yes	Yes	Yes	Yes	Yes	Yes	Yes	Yes	Yes	Yes	Yes
**Causality**														
4. Were other alternative causes that may explain the observation ruled out?	Yes	Yes	Yes	Cases 1–3: Yes Case 4: No	Yes	Yes	Yes	Yes	Yes	Yes	Yes	Yes	Yes	Yes
5. Was there a challenge/rechallenge phenomenon?	n/a	n/a	n/a	n/a	n/a	n/a	n/a	n/a	n/a	n/a	n/a	n/a	n/a	n/a
6. Was there a dose–response effect?	n/a	n/a	n/a	n/a	n/a	n/a	n/a	n/a	n/a	n/a	n/a	n/a	n/a	n/a
7. Was follow‐up long enough for outcomes to occur?	Yes	Yes	Yes	Yes	Yes	Yes	Case 1: No Case 2: Yes	Yes	Yes	Yes	No	Yes	Yes	Yes
**Reporting**														
8. Can others replicate the research or apply it to practice?	Yes	Yes	Yes	Yes	Yes	Yes	Yes	Yes	Yes	Yes	Yes	Yes	Yes	Yes

## RESULTS

Initial querying of the five databases yielded 404 articles. Detection of duplicates via Rayyan.ai's automatic function and subsequent removal yielded 178 original articles. Following duplicate removal, articles were then subjected to abstract and title appraisal. Out of the 178 articles, only 20 remained. Full‐text appraisal was then conducted on the 20 articles with 14 satisfying inclusion criteria. Patient data were then extracted from the included articles (Tables 4 and 5) and subjected to descriptive analysis (Table 6).

**Table 4:** Extracted data.

Author(s)	Title	Vaccine type	Number of cases
Bensasson^28^	A case of algodystrophic syndrome of the upper limb following tetanus vaccination	Tetanus	1
Al‐Nesf et al.^29^	Complex regional pain syndrome Type I following tetanus toxoid injection	Tetanus toxoid	1
Pirrung^30^	Complex regional pain syndrome (CRPS) after a local vaccination against hepatitis Type A	Hepatitis A	1
Jastaniah et al.^26^	Complex regional pain syndrome after hepatitis B vaccine	Hepatitis B	4
Genc et al.^31^	Complex regional pain syndrome Type‐I after rubella vaccine	Rubella	1
Kwun et al.^32^	Complex regional pain syndrome by vaccination: A case of complex regional pain syndrome after vaccination of influenza A(H1N1)	Influenza A	1
Martínez‐Lavín^27^	Fibromyalgia‐like illness in two girls after human papillomavirus vaccination	Quadrivalent HPV	2
Bilić et al.^33^	Complex regional pain syndrome Type I after diphtheria‐tetanus (Di‐Te) vaccination	Diphtheria–tetanus toxoid	1
Seyrek et al.^34^	Complex regional pain syndrome in an adult following tetanus‐diphteria [*sic*] toxoid vaccine: Case report and review of the literature	Diphtheria–tetanus toxoid	1
Koushik^35^	Complex regional pain syndrome in a young boy after receiving a diphtheria, tetanus, and acellular pertussis vaccine	DTaP	1
Raman,^25^	Complex regional pain syndrome post COVID‐19 vaccine shot: An autobiographical case report	COVID‐19 (unspecified type)	1
Cho et al.^38^	Post‐COVID‐19 vaccination arm pain diagnosed as complex regional pain syndrome: A case report	COVID‐19 (mRNA Pfizer)	1
Horisawa et al.^36^	Complex regional pain syndrome after mRNA‐based COVID‐19 vaccination	COVID‐19 (mRNA Pfizer)	1
Nogueira Pinto et al.^37^	Stellate ganglion block for complex regional pain syndrome treatment after SARS‐CoV‐2 vaccine: A case report	COVID‐19 (mRNA Moderna)	1

**Table 5:** Descriptive analysis.

Author(s)	Age (years)	Sex	Medical history	Time to symptom onset
Bensasson^28^	48	M	Diabetes requiring temporizing insulin therapy once in the past, abscess on buttocks at time of vaccination, Dupuytren's contracture	1 day after vaccination
Al‐Nesf et al.^29^	32	M	Former smoker	16 h after vaccination
Pirrung^30^	18	F	No pertinent history reported	2 h after vaccination
Jastaniah et al.^26^	1: 12; 2: 12; 3: 13; 4: 12	F (all)	1: Hashimoto's, 2: No pertinent history; 3: No pertinent history; 4: Localized swelling after DTaP vaccination	1: 1 h after vaccination; 2: 30 min after vaccination; 3: 30 min after vaccination; 4: 15 min after vaccination
Genc et al.^31^	11	F	Relationship problems with parents	20 min after vaccination
Kwun et al.^32^	17	F	Report patient was previously on medication (medication not specified)	7 h after vaccination
Martínez‐Lavín^27^	1: 11, 2: 14	F (all)	1: No pertinent history reported; 2: family history of spondyloarthropathy	1: Developed symptoms immediately after 2nd vaccine that resolved after 1 week, Developed lasting symptoms 2 days after 3rd vaccination; 2: 4 weeks after 2nd vaccination
Bilić et al.^33^	18	F	Laryngitis treated with antibiotics 3 weeks prior, severe emotional stress and sleep disorder for 1 year before vaccination, obstructive bronchitis until age 6, and allergies to dust and maggots	15 min after vaccination
Seyrek et al.^34^	26	F	No pertinent history reported, positive ANA titer (1:100) but negative ANA profile at diagnosis	Localized rash immediately after vaccination, CRPS symptoms 2 days later
Koushik^35^	10	M	No pertinent history reported	Within 24 h after vaccination
Raman,^25^	33	F	No pertinent history reported	Within 24 h of 1st vaccination in series
Cho et al.^38^	32	F	Hypothyroidism currently controlled with levothyroxine	3 h after unspecified dose in series
Horisawa et al.^36^	17	F	No pertinent history reported	2 weeks after 2nd vaccination in series
Nogueira Pinto et al.^37^	58	F	HLD, HTN, recent acute embolic stroke of undetermined source of the right carotid artery with striatocapsular and insular ischemia, and Takotsubo syndrome	24 h after 3rd vaccination in series

Abbreviations: ANA, antinuclear antibody; CRPS, complex regional pain syndrome; DTaP, diphtheria, tetanus, and pertussis [DTaP] vaccine, HLD, hyperlipidemia; HTN, hypertension.

**Table 6:** Descriptive analysis of complex regional pain syndrome reports.

	*n (%)*
No. of patients	18
No. of case reports	14
No. of case series	2
F	15 (83.3%
M	3 (16.6%)
	***n (%)***
No. of patients with autoimmune condition	1 (5.6%)
No. of patients with reaction to previous dose of same vaccine	2 (11.1%)
No. of patients with previous vaccine reaction unrelated to current vaccination	1 (5.6%)
	**Years**
Mean patient age	21.9
Median patient age	17
	**Time in hours (SD)**
Mean time to symptom onset	68.38 (169.35)
Median time to symptom onset	11.5

### Full‐text articles excluded from analysis

Of the articles subjected to full‐text appraisal, five were excluded from the final analysis. Four of the articles were the wrong publication type. This meant that the articles were either retrospective cohort studies, conference abstracts, posters, or any other study that was not a case report. One study was excluded because it was unable to be located through any means including but not limited to inter‐library loan.

### Results of quality of evidence rating and risk of bias assessment

Regarding GRADE scoring, all studies were rated as very low evidence quality, primarily due to their status as case reports or case series. Of note, Raman was noted to have a very serious risk of bias and was strongly suspected of publication bias due to its autobiographical nature.^25^

Regarding methodological quality for risk of bias, most studies answered “yes” to all questions. Studies that did not satisfy all assessments of bias are as follows. Jastaniah et al. did not adequately address alternative explanations in their report for Case 4 (a 12‐year‐old with a history of localized diphtheria, tetanus, and pertussis [TDaP] vaccination reactions).^26^ Martínez‐Lavín did not report follow‐up for Case 1 (an 11‐year‐old with no medical history reported).^27^ Raman did not report follow‐up.^25^

### Overview of outcomes

Of the included articles, a total of 18 patient cases were identified, spanning 1977 to 2023 (*n* = 18). Fifteen patients are reported to be female (83.3%) and three male (16.6%). Patient ages ranged from 10 years old to 58 years old. The average age at presentation was 21.9 years (median = 7 years). Eight patients did not report any pertinent medical history (44.4%), one case reported a history of autoimmune disease (Hashimoto's thyroiditis) (5.6%), one patient had had a recent infection treated with antibiotics (5.6%), and one patient reported a history of hypersympathetic activation (Takotsubo cardiomyopathy) and recent stroke (5.6%). Three patients reported non‐anaphylactic reactions to prior vaccinations (one with DTaP and two with the initial dose of a series), while 15 endorsed tolerating prior vaccinations well. It is worth noting that one patient did have a positive antinuclear antibody (ANA) titer of 1:100, but a negative ANA profile, which the researcher denoted as not having an autoimmune disease. The mean time to symptom onset was 68.38 h (SD = 169.35 h; median = 1.5 h).

### Tetanus vaccination

Of the 14 cases included in this paper, two focused on tetanus vaccinations. Time to symptom onset varied, but both were reported to be greater than 12 h. Bensasson et al. reported one case with a history of a 48‐year‐old male with non‐insulin‐dependent diabetes, Dupuytren's contracture, and a gluteal abscess at the time of vaccination.^28^ Al‐Nesf and Abdulaziz reported a 32‐year‐old male who was a former smoker.^29^

### Hepatitis B vaccine

Jastaniah et al. reported four young females who developed CRPS following hepatitis B vaccination.^26^ Three of the females were 12 and one was 13 years of age. The time to onset of symptoms ranged from 15 to 45 min, with an average time to onset of 33.7 min. Three of the girls had tolerated previous vaccinations well, with one having a localized reaction to TDaP in the past. Only one of the girls had a history of autoimmune disease, being previously diagnosed with Hashimoto's at the age of 10.

### Hepatitis A

Pirrung reported the case of an 18‐year‐old female developing CRPS symptoms 2 hours following hepatitis A vaccination injection.^30^ She was not reported to have had any prior pertinent medical history.

### Rubella vaccine

Of the 14 case reports included, there was only one discussing initial CRPS symptoms following rubella vaccination. Genc et al. reported an 11‐year‐old female with no pertinent medical history, who developed CRPS symptoms 20 min post‐injection and was subsequently diagnosed with CRPS Type 1.^31^

### Influenza vaccine

Kwun et al. described the case of a 17‐year‐old female who developed CRPS following influenza A (H1N1) vaccination.^32^ The patient was reported to have previously been on medication but no specific pharmacologic name is given. She developed her first symptoms 7 hours post‐injection.

### Tetanus–diphtheria containing vaccines

Three case reports reported on tetanus–diphtheria‐containing vaccinations. Bilić et al. examined the case of an 18‐year‐old female who developed symptoms 15 min after tetanus–diphtheria vaccination.^33^ The patient had a history of obstructive bronchitis until age six, allergies to dust and maggots, high emotional stress levels, a sleeping disorder, and a recent bout of laryngitis treated with antibiotics. The following year, Seyrek et al. reported the case of a 26‐year‐old female, who developed a rash immediately after injection with a tetanus–diphtheria toxoid vaccine, and CRPS symptoms 2 days later.^34^ The patient is said to have tolerated prior vaccinations but did have a positive ANA titer of 1:100 with a negative ANA profile. Lastly, Koushik reported a 10‐year‐old male who developed CRPS symptoms 24 h following injection with a DTaP vaccine.^35^ He was said to have no prior medical history of note.

### COVID‐19 vaccination

Most recently were four cases reporting CRPS following COVID‐19 vaccination. All of the case reports were about women ranging in age from 17 to 58 years. Horisawa et al. reported a 17‐year‐old patient on the second dose of the Pfizer series with no pertinent medical history with symptoms beginning 24 h after the second vaccination in the series.^36^ Nogueira Pinto et al. reported a 58‐year‐old female on the third shot of the Moderna series with a recent stroke with subsequent Takotsubo cardiomyopathy as well as a history of hypertension and dyslipidemia whose symptoms onset occurred 2 weeks after the second vaccination in the series.^37^ Cho et al. reported a 32‐year‐old patient who was on an unspecified dose of the Pfizer series with a history of hypothyroidism controlled with levothyroxine whose symptoms onset occurred within 3 hours of administration.^38^ Raman reported a 33‐year‐old female receiving an unspecified version of the COVID‐19 vaccine with no pertinent medical history reported whose symptoms onset occurred within 24 h of the first vaccination in the series.^25^

### HPV vaccination

Of the 14 included case reports, Martínez‐Lavín studied two cases after HPV vaccination in 2014 focusing on two females.^27^ An 11‐year‐old developed symptoms immediately after her second vaccination that lasted for 1 week who then developed lasting symptoms 2 days after the third vaccination. A 14‐year‐old with a family history of spondyloarthropathy developed symptoms 4 weeks after the second vaccination.

## DISCUSSION

We report the most up‐to‐date systematic review of case reports on this topic; yet, as of November 2023, there is still no consensus as to the relationship between CRPS and vaccine administration.

The largest numbers of cases were equally reported with the hepatitis B vaccination with four cases reported in a single case series and with the SARS‐CoV‐2 mRNA vaccination, where four cases were reported (two with the Pfizer version, one with the Moderna version, and one was unspecified). The smallest numbers of cases were reported for influenza (H1N1), rubella, and hepatitis A vaccination, each with one case reported and included in the analysis. The increased prevalence of females with CRPS in the included case reports after any immunization is in accordance with the established propensity for females to develop CRPS at rates higher than males.^38^ Four of the 18 cases had no pertinent medical history reported therein (22.2%).

### Factors contributing to development of CRPS

CRPS is accepted to be multifactorial but is still not well understood. Often linked to an inciting event, greater than 60% of cases are traumatic in nature, such as surgery, a fracture, crush injury, or a similar type of injury that precipitates the characteristic autonomic dysfunction and neuropathic pain.^39^ Immune dysfunction (increases in proinflammatory markers and decreases in anti‐inflammatory markers) is a hallmark of the risk of development of CRPS.^27^ The immunological principle behind vaccination lies in stimulating an immune response to engage active immunity against various microbial and viral illnesses. While it is theoretically possible the immunologic response from vaccination may be sufficient to trigger the development of CRPS, a recent retrospective study investigating the prevalence of CRPS after HPV vaccination found rates of development of CRPS were no higher than general population rates of CRPS (incidence rate in study: 4.35–5.94 per 100,000 person‐years vs. general population incidence: 5.5–26.2 per 100,000 person‐years).^40^ This finding further supports the global safety of vaccines as it relates to CRPS risk.

While none of the cases reported a concern for, or active diagnosis of, somatic symptom disorder, somatoform symptom disorders should be considered in cases of Type 1 CRPS. There is scant data assessing the possible overlap and rates of comorbidity or misdiagnosis in these conditions. There is a risk of misdiagnosis as the presentations can be similar, though the etiologies differ vastly.^21^ A review of 50 CRPS patients undergoing psychiatric testing for medicolegal purposes found that 42% had a history of at least two pain‐related somatic syndromes.^39^

It is possible that these individuals who are diagnosed with CRPS after vaccination may have already been at significantly increased risk for the development of CRPS and thus the inflammatory nature of immunization started the pathological process of central sensitization and autonomic dysfunction. For an unknown reason, immune cells can exhibit a bidirectional relationship with neuroinflammation in the development and propagation of CRPS.^41^ Only one included case was reported in a former smoker which could contribute to vascular inflammation depending on their time since abstaining. The presence of one autoimmune disease (Hashimoto's case) and one reported localized vaccine adverse event to a specific immunization which was not the subject of the case report suggests a lack of clinically meaningful and direct evidence of prior immunologic or inflammatory dysfunction related to CRPS development. Live vaccines produce the more potent immunogenic response and thus the most potent and long‐lasting immune memory.^42^ The lack of a high number of cases with live vaccines instills safety behind the fundamental principles of immunization and demonstrates a qualitatively low risk of CRPS after immunization.

Psychosocial stress is frequently cited as a predisposing factor in the development of CRPS; however, this association has not been fully borne out in research. A retrospective study of children with CRPS found that these children self‐reported lower rates of anxiety and depression than their cohorts with abdominal pain, though the CRPS patients did experience more stressful family‐related life events overall and experienced more stressful life events before diagnosis than children with chronic headaches.^43^ A retrospective study using the Minnesota Multiphasic Personality Inventory‐2 to compare depressive symptoms in CRPS patients to their major depressive disorder (MDD) cohort found that patients with CRPS had some higher subscale scores than controls but scored lower than MDD patients overall.^44^ One prospective study examining post‐traumatic stress disorder (PTSD) in CRPS patients found a significantly higher prevalence of PTSD in patients with CRPS that was predominately diagnosed before CRPS developed.^45^ There is a lack of research comparing CRPS patients with healthy patients and a lack of prospective studies on CRPS. Overall, the link between psychosocial stress and CRPS is still theoretical. One case demonstrated significant psychosocial stress at baseline and another case reported relationship problems with parents. Two patients were described as having a fibromyalgia‐like condition but were ultimately given the diagnosis of CRPS. Both depression and fibromyalgia share mechanisms such as central sensitization.^46^ These shared pathways could contribute to the development of CRPS but little is known about CRPS in patients with fibromyalgia.

Genetic factors may also play a role, but there is still no convincing and definitive evidence regarding specific genes and genetic factors.^40^ Included studies did not address genetic factors associated with CRPS, however, only one case had a family history of spondyloarthritis but did not confirm the presence of the HLA‐B27 haplotype. Patients with spondyloarthritis have a high prevalence of chronic pain but not necessarily CRPS.^47^

Finally, alterations in plasma catecholamines are noted in patients with CRPS. There have been observed decreases in plasma norepinephrine in the affected upper extremity even in the context of chronic CRPS and cutaneous vasoconstriction.^40^ There was no confirmatory evidence of catecholamine alterations in the included cases.

### Limitations

As with any study, this particular study had limitations specific to its design and objectives. As this was a systematic review of case reports and case series, this study did not include high‐quality controlled studies or retrospective studies assessing the prevalence of CRPS. Vaccine Adverse Event Reporting System (VAERS) data were not queried for the purposes of this study, possibly excluding instances of CRPS reported by trained health professionals or patients themselves. Individuals were not excluded based on past medical history as this contributes to the multifactorial pathophysiology of this syndrome but could contribute to bias in assessing the influence of vaccination on CRPS development. Symptoms at onset or discharge were not included in this paper as the goal was not to evaluate the accuracy of CRPS diagnosis. Overall, the GRADE for the 14 articles included in this systematic review was very low in quality as the studies were case reports of non‐randomized, uncontrolled events. Grey literature was excluded from analysis on the basis of heterogenous search strategies, desire to exclude unpublished reports, dissertations, and conference abstracts as the primary interest of this investigation was that of published and peer‐reviewed case reports. The exclusion of such literature may lead to the omission of potentially meaningful data, such as unpublished clinical trial data.

### Future directions

While completing this systematic review, areas of future research and investigation were uncovered. VAERS data from verified healthcare professionals reporting CRPS could prove useful in determining the prevalence or potential risk of CRPS associated with various routine vaccinations. The exact pathophysiology of this syndrome is still unknown, but there are known contributing factors and mechanisms associated with its development. Immune dysfunction is a commonly recognized component of CRPS progression. Further investigation into how vaccinations can contribute to the development of CRPS in certain populations could prove useful in further elucidating the underlying pathologic mechanisms behind CRPS and its presentations.

## CONCLUSION

Through our systematic review, we were able to analyze the current literature and further the understanding regarding the development of CPRS as an adverse effect of vaccine administration. The limitations regarding the quality of evidence used in this study highlight the need for a greater output of higher‐level evidence in the form of controlled trials and large‐scale retrospective studies to help further elucidate the connection between vaccine use and CRPS development. CRPS is a complex, multifactorial condition wherein the exact pathophysiology currently remains unclear. Furthermore, exploring the science behind the development of CRPS and analyzing patients with CRPS for potential risk factors and epidemiological factors will better help develop management and ultimately preventative plans of care. At the time of this paper, vaccines continue to be safe for global public use as they relate to CRPS.

## AUTHOR CONTRIBUTIONS

William K. Copenhaver oversaw the project, wrote the results section, edited the paper, and performed data extraction. Brandon J. Goodwin completed the literature retrieval and the methods section and performed data extraction. Alexa Simonetti and Kunal P. Shah wrote the introduction and constructed tables. Nicholas J. Averell wrote the conclusion section, the abstract, and performed data extraction. David F. Lo provided significant edits to the entire paper. Richard T. Jermyn edited the paper and oversaw the project.

## CONFLICT OF INTEREST STATEMENT

The authors declare no conflict of interest.

## ETHICS APPROVAL STATEMENT

All applicable ICMJE ethical standards were strictly followed.

## PATIENT CONSENT STATEMENT

As no human participants were recruited, no consent was required or obtained in the production of this manuscript. No clinical trial registry was pursued due to lack of human participant registration.

## CLINICAL TRIAL REGISTRATION

N/A.

## Data Availability

All data produced and obtained in the production of this manuscript are either represented in this manuscript or are available in the references statement.
